# Seroprevalence and Epidemiology of *Toxoplasma gondii* in Animals in the Qinghai-Tibetan Plateau Area, China

**DOI:** 10.3390/pathogens10040432

**Published:** 2021-04-06

**Authors:** Guojing Li, Wangli Zheng, Jinfang Yang, Tongsheng Qi, Yongcai He, Wangkai Chen, Hejia Ma, Yali Sun, Ying Li, Ming Kang, Jixu Li

**Affiliations:** 1 State Key Laboratory of Plateau Ecology and Agriculture, Qinghai University, Xining 810016, China; liguojing@qhu.edu.cn (G.L.); wanglizheng@qhu.edu.cn (W.Z.); yangjinfang@qhu.edu.cn (J.Y.); qitongsheng@qhu.edu.cn (T.Q.); heyongcai@qhu.edu.cn (Y.H.); chenwangkai@qhu.edu.cn (W.C.); mahejia@qhu.edu.cn (H.M.); yalisun@qhu.edu.cn (Y.S.); 2000990008@qhu.edu.cn (Y.L.); 2013990003@qhu.edu.cn (M.K.); 2 College of Agriculture and Animal Husbandry, Qinghai University, Xining 810016, China

**Keywords:** *Toxoplasma gondii*, toxoplasmosis, ELISA, IgG, IgM, animals, Qinghai-Tibetan Plateau Area

## Abstract

*Toxoplasma gondii* belongs to the Apicomplexan protozoa—an obligate intracellular parasite—causing toxoplasmosis that has a worldwide distribution and is very harmful to both human health and the livestock industry. However, the information on toxoplasmosis in the Qinghai-Tibetan Plateau Area (QTPA) and the seroprevalence of *T. gondii* in the food-borne animals in that area has been limited. Therefore, this study focused to *T. gondii* and toxoplasmosis to perform an indirect ELISA test based on recombinant *Tg*SAG2 protein to establish a comprehensive record of the seroprevalence of *T. gondii* infections in a wide range of animals, including Tibetan sheep (*Ovis aries*), yaks (*Bos grunniens*), cows, chicken, pigs, and horses, in the QTPA. Overall, the seropositive rates of the specific-*T. gondii* IgG and IgM antibodies in all investigated animals were 44.1% (1179/2673) and 18.0% (469/2612), respectively. The 14.9% (389/2612) sera were determined to be both IgG and IgM positive samples, 30.2% (789/2673) were single-IgG seropositive, and a total of 80 in 2612 animals (3.0%) were single-IgM seropositive. Moreover, for the animal species, the pig was the most prevalent animal (90.2%, 304/337) for IgG positivity, followed by Tibetan sheep (50.7%, 460/907), chickens (45.8%, 229/500), yaks (21.1%, 140/663), cows (18.5%, 38/205) and horses (13.1%, 8/61), respectively. For the IgM antibody positivity, the pig was also the most prevalent animal (41.8%, 141/337), followed by Tibetan sheep (21.2%, 191/907), cows (15.1%, 31/205), chickens (12.4%, 62/500) and yaks (6.6%, 44/663), respectively. The significant differences in the prevalent distribution of *T. gondii* were found in the different altitudes. In conclusion, this study found the high seroprevalence for *T. gondii* infections among these animal species in the QTPA, and provides new data to facilitate further research for development of control measures against *T. gondii* infections in the surveyed locations.

## 1. Introduction

The Qinghai-Tibetan Plateau Area (QTPA), the largest plateau with the highest average altitude on the planet, is located in northwestern China [1]. The Qinghai province, located on the northeastern side of QTPA, has a unique and vigorous natural ecosystem due to the high altitude (an average elevation of more than 2000 m above sea level) and cold climate (an average annual temperature below 10 °C) [2,3]. Therefore, a variety of unique livestock has been domesticated in the province, including Tibetan sheep (*Ovis aries*), yaks (*Bos grunniens*), cows, chicken, pigs, and horses [4], which are the important economic livestock animals and food animals in this special area [5]. These animals share water and food in the plateau area [6,7]. 

*Toxoplasma gondii* is a very important food-borne zoonotic pathogen that can infect almost all warm-blooded animals, including humans, livestock and birds [8,9,10,11,12,13,14] and also causes economic losses in the livestock industry [15,16,17]. Tibetan sheep and yaks are indigenous species raised under extensive animal systems, while cows, chickens, pigs, and horses are important economic livestock animals in the QTPA. The animals could be the intermediate hosts of *T. gondii* in this plateau area. Humans or these animals might be infected by ingesting water and foods which contain infecting oocysts from cats to develop toxoplasmosis [8,14,16,17]. Therefore, this study focused on *T. gondii* and toxoplasmosis to investigate the seroprevalence of *T. gondii* IgG and IgM antibodies in a wide range of animals in the QTPA.

The serological tests play a crucial role in the diagnosis of toxoplasmosis and were widely used [18]. In China, some studies have been reported that humans and animals were infected by *T. gondii* with different prevalence, of which the prevalence in food animals (such as cattle, sheep, goat, chickens, and swine) was higher than that in humans [19,20]. Although there are several reports on the seroprevalence and epidemiology of *T. gondii* in animals from the different regions in the QTPA, most attempts of testing toxoplasmosis have been done in yaks which are the important economic animals and food-borne animals in the QTPA [21,22,23,24,25,26]. To date, information on toxoplasmosis in the QTPA and the seroprevalence of *T. gondii* in other food animals in that area has been limited. 

Among several serodetection tests used for diagnosis of toxoplasmosis, the latex agglutination test (LAT) and indirect haemagglutination (IHA) were shown to be insensitive in their present form, the indirect fluorescent antibody test (IFAT) and modified agglutination test (MAT) are considered time-consuming and expensive though specific; hence, the indirect ELISA test has been accepted as the most practical test especially for a large number of samples [9,11]. The surface antigen 2 (SAG2) of *T. gondii* has an added diagnostic relevance, as it is exposed to the immune system of the hosts [18], and has been identified and tested as an important candidate for the serological diagnosis for toxoplasmosis in many studies [27]. Furthermore, the ELISA based on recombinant SAG2 fusion protein has been used as an effective field serodiagnostic test for the detection of *T. gondii* infection in different animal species, such as cat, cattle, sheep, goat and pig [18]. Thus, the aim of this study was to perform an indirect ELISA test based on recombinant *Tg*SAG2 protein (r*Tg*SAG2) to establish a detailed record of the seroprevalence of *T. gondii*-specific IgG and IgM antibodies in Tibetan sheep, yaks, cows, chicken, pigs, and horses in the QTPA.

## 2. Results

**Figure 1:**
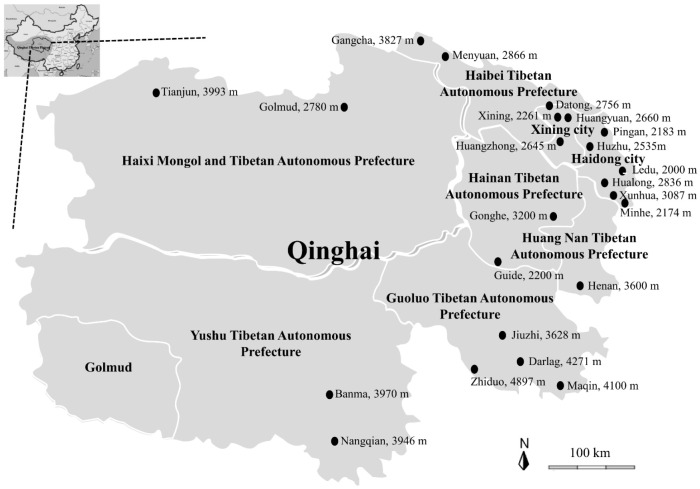
The map of the Qinghai-Tibetan Plateau Area and Qinghai province, showing the name of sampling sites and the height above sea level of sampling site per sampling site included. The Tibetan sheep, yak, cow, chicken, pig, and horse serum samples were collected at two cities and six prefectures of the Qinghai province indicated by the black circle. The figure was generated and modified using GIMP 2.8.10 (gimp-2.8.22-setup, https://www.gimp.org/, accessed on 20 February 2021).

In this study, the seroprevalence of *T. gondii* in the animals from the QTPA (Figure 1, Table 1), including Tibetan sheep, yaks, cows, chickens, pigs, and horses, was investigated by the indirect ELISA.

**Figure 2:**
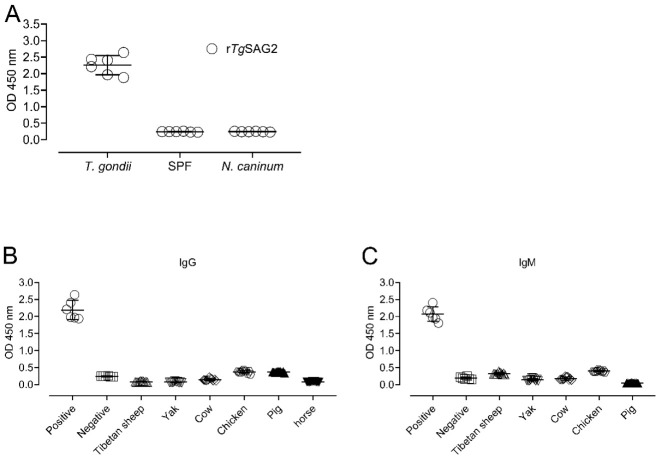
The confirmation of the r*Tg*GRA7-based indirect ELISA. (**A**) The positive and negative serum samples of mice for *T. gondii* or the positive serum samples of mice for *N. caninum* were used to develop the ELISA assay. (**B**,**C**) The cut-off values for the positive samples of *T. gondii* IgG (**B**) and IgM (**C**) were determined by using negative controls of Tibetan sheep, yaks, cows, chickens and pigs, respectively.

To confirm the r*Tg*SAG2-based indirect ELISA in this study, the positive and negative serum samples of mice for *T. gondii* or the positive serum samples of mice for *N. caninum* were used to develop the ELISA assay. The results showed that r*Tg*SAG2 protein could determine *T. gondii* positive sera but no any reaction with *T. gondii* negative or *N. caninum* sera (Figure 2A), suggesting current recombinant protein could be studied with animal sera for detecting the *T. gondii* specific-antibody. Moreover, the cut-off value for judging positive samples was determined by using negative controls. The cut off values were determined as 0.120, 0.114, 0.246, 0.482, 0.423 and 0.110 for IgG antibodies based on r*Tg*SAG2-ELISA in Tibetan sheep, yaks, cows, chicken, pigs, and horses; and 0.411, 0.234, 0.306, 0.468 and 0.065 for IgM antibodies based on r*Tg*SAG2-ELISA in Tibetan sheep, yaks, cows, chickens and pigs, respectively (Figure 2B,C).

In this study, a total of 2673 animal serum samples were tested in a detailed survey of specific anti-*T. gondii* IgG antibodies in different sampling sites among Tibetan sheep, yaks, cows, chickens, pigs, and horses, and the 2612 animal serum samples were investigated the specific anti-*T. gondii* IgM antibodies in different areas among Tibetan sheep, yaks, cows, chickens and pigs from Qinghai. The overall seropositive rates of IgG and IgM in all investigated animals were 44.1% (1179/2673) and 18.0% (469/2612), respectively (Table 2). Moreover, as shown in Table 3, the 47.1% (1258/2673) animals showed at least one *T. gondii* specific-IgG or IgM was positive. Analysis of the positive animals, showed that the 14.9% (389/2612) sera were determined to be both IgG and IgM positive samples, 30.2% (789/2673) were single-IgG seropositive, and a total of 80 in 2612 serum samples (3.0%) were single-IgM seropositive which contains 3.7% (34/907), 2.0% (14/663), 8.8% (18/205), 0.6% (3/500) and 3.3% (11/337) single-IgM positive animals for Tibetan sheep, yaks, cows, chickens and pigs, respectively (Table 3).

**Figure 3:**
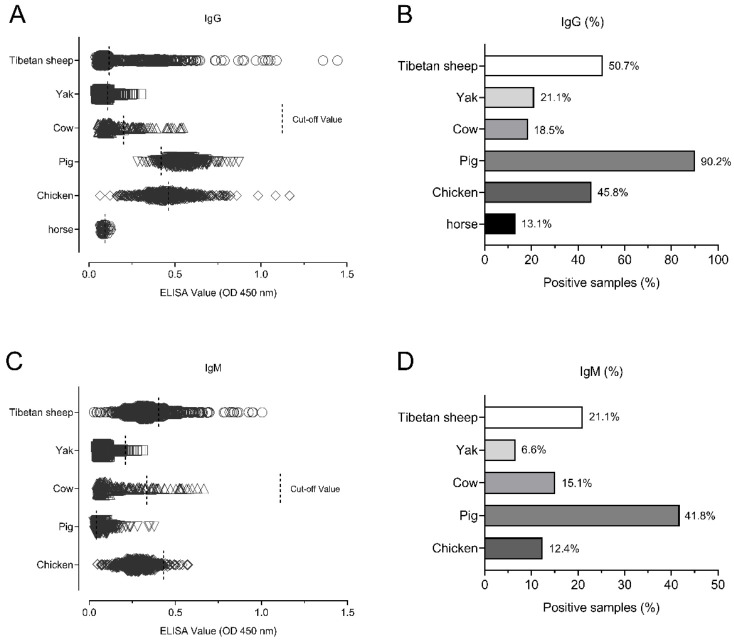
The seroprevalence among different animals. (**A**,**B**) The seroprevalence for IgG positivity in the Tibetan sheep, yaks, cows, chickens, pigs, and horses. (**C**,**D**) The seroprevalence for IgM positivity in the Tibetan sheep, yaks, cows, chickens, pigs, and horses.

The seroprevalence among the different animal species was tested and analyzed. Of the animal samples, the pig was the most prevalent animal (90.2%, 304/337) for IgG positivity, followed by Tibetan sheep (50.7%, 460/907), chickens (45.8%, 229/500), yaks (21.1%, 140/663), cows (18.5%, 38/205) and horses (13.1%, 8/61) (Figure 3A,B). For the IgM antibody positivity, the pig was also the most prevalent animal (41.8%, 141/337), followed by Tibetan sheep (21.2%, 191/907), cows (15.1%, 31/205), chickens (12.4%, 62/500) and yaks (6.6%, 44/663) (Figure 3C,D), respectively. 

To analyze the effect of altitude in the seroprevalence, all serum samples were divided into three groups, 2000–3000, 3000–4000, and 4000–5000 m (Table 4). As shown in Table 4, the analyzed results showed that the *T. gondii* IgG and IgM seroprevalence among the altitude groups was significantly different (*p* < 0.05). Moreover, the seroprevalence of IgG and IgM significantly differed among animal species (*p* < 0.05). 

## 3. Discussion

Tibetan sheep, yaks, cows, chickens, pigs, and horses play an important role in the agricultural economy in the QTPA, and are the main food-borne animals. Tibetan sheep, famous for their high-quality pelage and their nutritious delicious meat, are widely distributed in Qinghai. The majority of yaks in the world are living on the QTPA, while approximately 35.0% (~4.9/14 million of the yaks) of the yaks in a total of the QTPA are distributed in Qinghai province [22]. Toxoplasmosis has a wide distribution in this plateau area and is very harmful to both human health and the livestock industry. The current study provides a valuable note of the serological prevalence of *T. gondii* IgG antibodies and the first record of epidemiology of *T. gondii* IgM antibodies in all these surveyed animals on the whole Qinghai province, which contains two cities and six prefectures.

Since the indirect ELISA test has been accepted as the most practical test in the diagnosis of toxoplasmosis especially for a large number of samples, it has been used for seroprevalence and epidemiology of *T. gondii* in humans and animal species such as cat, cattle, sheep, goat and pig [18,27]. Therefore, in this study, we choose the indirect ELISA method to sero-detect the antibodies of *T. gondii* in sera samples from different animal species. The SAG2-based ELISA, presents its immunodominant nature, could determine *T. gondii*-specific antibodies with the high sensitivity from both acute and chronic *T. gondii* infection when used either alone or in combination with the other proteins. Importantly, the recombinant SAG2 protein has adequate diagnostic sensitivity and specificity for both *T. gondii*-specific IgG and IgM antibodies without cross reactivity [28]. Hence, all these special characteristics could make r*Tg*SAG2 a promising candidate for the sero-diagnosis of *T. gondii* IgG and IgM antibodies in the different animal species in this study. Our study confirmed that current indirect ELISA based on r*Tg*SAG2 not only detected the specific-*T. gondii* IgG and IgM antibodies but also distinguished them well.

In the current study the seroprevalence of *T. gondii* infection in Tibetan sheep was 50.7% (460/907). Previous studies showed a lower seroprevalence of *T. gondii* infection in Qinghai Tibetan sheep (29.8%), evaluated by indirect hemagglutination (IHA) test [23]. In the other two equally important provinces in QTPA, Gansu, and Tibet, adjacent to Qinghai, the seroprevalence results were also lower. In Gansu province was found a seroprevalence of 20.3%, evaluated by modified agglutination test (MAT) [29], and in Tibet province, a seroprevalence of 5.7% was found (26/455), using an IHA [26]. Although limited in current information, testing technologies, and the number of samples, our data indicate the epidemiological prevalence of *T. gondii* infection in Tibetan sheep with a high rate in Qinghai in the QTPA. *T. gondii* seroprevalence in sheep and goats varies with altitude and climate, and sheep and goats from the eastern coastal locations have a higher prevalence rate than that of sheep from the west plateau part in China [21]. However, the opposite is that for this plateau organism, we found the highest infection rate in the highest altitude in this study (Table 4). Therefore, the prevalence of pathogens will change with the changes in the living environment of animals, especially in the plateau environment. Moreover, the high prevalence of *T. gondii* infection in Tibetan sheep may be due to not only oocyst original infection but also transplacental transmission.

*T. gondii* could indirectly infect humans through ingesting the undercooked or raw meat or milk from yaks or cows. Although cattle are not considered to be an outstanding host for *T. gondii* [21], the positive rates of 21.1% or 18.5% for *T. gondii* IgG antibody and 6.6% or 15.1% for *T. gondii* IgM antibody in yaks or cows were found in this study, respectively. Yak is the main meat consumed by humans in Qinghai, while the cow is the source of milk for humans in the tested area [30]. The current seroprevalence of *T. gondii* in yaks or cows and the living habits (such as some ethnic groups that consume raw or undercooked meat or with milk) in the QTPA, not only cause economic losses, but also seriously threatens the local human health. Furthermore, the seroprevalence of *T. gondii* infection in the current study in yaks from Qinghai province was consistent with the average prevalence of *T. gondii* infection, which was 20.8% ranging from 8.3 to 26.4% in this area in 2008–2014 [30,31,32,33]. This suggests steady epidemiology of *T. gondii* in yaks in this plateau area. The higher seroprevalence of *T. gondii* in yaks may be due to geographical factors, such as rivers, foods, and wild animals that these animals share in the large ecological environment.

The current study was the first report for the seroprevalence and epidemiology of *T. gondii* in chickens, pigs and horses in the Qinghai, though these animals infected with *T. gondii* have been investigated in other province in China [25,34,35]. Of the investigated animal species in this study, the most prevalent animal for *T. gondii* infections was the pig, for which seroprevalence was significantly higher than other animals. Moreover, the current seroprevalence in pig showed significantly higher than that of previously reported in Shanxi, Jilin, Chongqing, and Tibet provinces in China [34,36,37,38]. These differences might be due to different climates and rearing systems. More important is that the contribution of cats and rodents could not be ignored in *T. gondii* infections [34]. Although the high levels of antibodies to *T. gondii* were not accompanied by severe clinical symptoms and high mortality in pigs in the current study, it could not be ignored that a serious toxoplasmosis outbreak in the neighboring province (Gansu province) led to pig deaths [39]. Therefore, the high seropositivity of IgG and IgM in pigs found in this study should attract more attention to prevent the outbreak of toxoplasmosis in the investigated area. In addition, the current seroprevalence of *T. gondii* in chickens was tested to be significantly higher than the average prevalence of *T. gondii* infection from 2000 to 2017 in China [21]. Generally, the chicken might be used as an indicator of environmental and soil contamination with the *Toxoplasma* oocysts because chickens could ingest the oocysts from soil or environmental food causing *T. gondii* infections [40]. Therefore, the local peoples should pay attention to the soil and environment polluted with *Toxoplasma* oocysts. Furthermore, the current ELISA test based on r*Tg*SAG2 found a slightly lower seroprevalence of *T. gondii* in horses (13.1%, 8/61) than the average seroprevalence of *T. gondii* infection (18.0%, 615/3413) in China [35]. Although this study was limited to the number of the horse samples, the high prevalence suggests the horses should not ingest food and water that is probably contaminated by the oocysts of *T. gondii* that might reduce the seroprevalence.

In this study, our data demonstrated all tested animal species showed a positive for *T. gondii* infection with the high prevalence of IgG and IgM antibodies in two cities and six prefectures of Qinghai, suggesting that the distribution of toxoplasmosis is ubiquitous across Qinghai. Moreover, analysis of the seroprevalence of *T. gondii* IgG and IgM in the tested animals from the 44 sampling sites were conveniently chosen from 2174 m to 4897 m altitudes, suggesting the significant differences present in the infection rates at different altitudes. This may be due to the high-altitude areas in grazing areas where exist the possibility of sharing common water and food among animals, while in the low-altitude areas are a large number of activities for humans, and definitive-host cats leading to these food-borne animals are frequently exposed to the infecting source, causing *T. gondii* infections.

Here, we investigated the seroprevalence of the specific-*T. gondii* IgG and IgM antibodies. The diagnosis of acute *T. gondii* infection depends on the detection of *Toxoplasma*-specific IgG and IgM antibodies. The IgG avidity test and IgM analysis are considered to be suitable methods for determining acute infections [28]. The current results showed that all tested animal species present the single-IgG positive animals, both IgG and IgM positive animals, and single-IgM positive animals (Table 3). Considering that IgM is a *T. gondii*-specific antibody that appears in the early stage of acute *Toxoplasma* infection, and as the infection progresses, IgG and IgM antibodies will be observed together, while the IgM will reduce and IgG will keep the high level in the later stage of the infection, and the infection will turn into a chronic *T. gondii* infection. Therefore, our study attests that these animals from the different altitude sampling areas tested are suffering from acute or chronic *Toxoplasma* infection or have become carriers of *T. gondii* antibodies after the infections. To the best of our knowledge, this study is the first record of the epidemiology of *T. gondii* IgM antibodies and distinguishes *T. gondii*-specific IgG and IgM antibodies in these food-borne animals in the Qinghai province and in China.

## 4. Materials and Methods

### 4.1. Sample Collection of Tibetan Sheep (Ovis aries), Yaks (Bos grunniens), Cows, Chickens, Pigs and Horses

In this study, a total of 2673 serum samples were collected from the different animals, and the 44 sampling sites were conveniently chosen from 2174 m above sea level to 4897 m in the two cities and six prefectures of the QTPA from June to November 2020 (Figure 1), including apparently healthy Tibetan sheep (n = 907), yaks (n = 663), cows (n = 205), chickens (n = 500), pigs (n = 337), and horses (n = 61) (Table 1). All procedures were carried out according to the ethical guidelines of Qinghai University.

### 4.2. Serum Harvest

These animal blood samples were kept in an icebox, then sent to the State Key Laboratory of Plateau Ecology and Agriculture, Qinghai University. The samples were centrifuged at 5000 rpm for 10 min, 4 °C to separate and harvest sera and stored at −20 °C until used.

### 4.3. Recombinant Protein Expression

The recombinant *Tg*SAG2 was expressed with previously described methods [27], with slight modifications. The concentration of r*Tg*SAG2 protein was measured with a bicinchoninic acid protein assay kit (Thermo Fisher Scientific, Inc., Rockford, IL, USA).

### 4.4. Indirect ELISA

The 96-well ELISA plates were coated with 1 μg/ml r*Tg*SAG2 protein diluted in coating buffer (0.05 M Carbonate-Bicarbonate, pH 9.6) and incubated at 4 °C overnight. The ELISA plates were washed by PBS-T (0.05% Tween-20) three times, and then blocked with 3% skimmed milk, then washed once. Collected sera were diluted by 1:100 and incubated for 1 h at 37 °C. The plates were washed with PBS-T six times. Then, the HRP conjugated anti-IgG or IgM secondary antibodies of the corresponding species (Bethyl Laboratories, Montgomery, TX, USA), were diluted 1:4000 for the Tibetan sheep, yak, cow, chicken, pig and horse sera, and added and incubated for another 1 h at 37 °C. After washing six times, TMB, (3, 3′, 5, 5′-Tetramethylbenzidine) substrate containing 2.5% H_2_O_2_ was used to develop the reaction for 20 min, and 50 μL stop solution (2 M sulfuric acid) was added to each well to stop the action of horseradish peroxidase in the substrate. The results were measured at OD 450 nm. The cut-off point was calculated as the mean values of OD 450 nm for standard *Toxoplasma*-negative sera kept in our laboratory (ten samples of each animal) plus three times the standard deviations of OD450 values of these negative controls. The positive and negative serum samples of mice for *T. gondii* (kept at in our Lab) or the positive serum samples of mice for *N. caninum* (gift from Prof. Lijun Jia from Yanbian University, Jilin, China) were set as control to confirm the r*Tg*SAG2-based indirect ELISA.

### 4.5. Statistical Analysis

To graph and analyze the data, GraphPad Prism 8 software (GraphPad Software Inc., USA) was used. The prevalence and 95% confidence intervals per pathogen species were calculated using the OpenEpi program (https://www.openepi.com/Proportion/Proportion.htm, accessed on 15 November 2020). The chi-squared test and the logistic regression analysis were used to compare proportions of detected sample positivity in different regions and among different animals. The differences were considered to be statistically significant when the resulting *p*-values were lower than 0.05.

## 5. Conclusions

This study demonstrates the high seroprevalence for the specific-*T. gondii* IgG and IgM antibodies among Tibetan sheep, yaks, cows, chickens, pigs, and horses in the QTPA. These give the new valuable data on the epidemiology of *T. gondii* in the plateau area, and suggest that we should pay more attention to improve the animal’s survival environment, provide more water sources, avoid exposure to the cat, improve animal welfare, and strengthen future prevention and control of *T. gondii* infection in these food-borne animals in this region. However, the current study is limited to the range of sampling points, the number of samples, and the lack of records for the definitive host cat’s activity around the sampling point; thus, the serological prevalence of *T. gondii* infections should likewise be extensively investigated in animals of food-borne and economic importance, including cats and other meat-producing animals. Future studies should assess the epidemiology of *T. gondii* in these local animal species.

## Figures and Tables

**Table 1:** The sampling sites of the different animal species in Qinghai province in this study.

Prefectures	Sampling Sites	No. of Serum Samples *						
		Tibetan Sheep	Yak	Cow	Chicken	Pig	Horse	Total
Haibei	Gangca	75	0	0	0	0	0	75
	Menyuan	60	20	0	0	0	0	80
Hainan	Gonghe	60	20	0	0	0	0	80
	Guide	32	0	0	0	0	0	32
Haixi	Golmud	60	20	0	0	20	0	100
	Tianjun	30	0	0	0	0	0	30
Yushu	Zhiduo	60	20	0	0	0	0	80
	Nangqian	52	0	0	0	0	0	52
Guoluo	Jiuzhi	45	66	0	0	0	0	111
	Maqin	152	110	0	0	0	0	262
	Darlag	141	103	0	0	0	0	244
	Banma	0	132	0	0	0	0	132
Xining	Xining	0	0	0	50	50	0	100
	Datong	40	119	0	50	0	0	209
	Huangyuan	0	0	0	50	47	0	97
	Huangzhong	0	0	0	50	48	0	98
Haidong	Ledu	60	20	0	50	72	0	202
	Pingan	0	0	0	50	50	0	100
	Huzhu	0	0	0	50	50	0	100
	Minhe	0	0	205	50	0	0	255
	Hualong	0	0	0	50	0	0	50
	Xunhua	0	0	0	50	0	0	50
Huangnan	Henan	40	33	0	0	0	61	134
Total		907	663	205	500	337	61	2673

* No.: Number.

**Table 2:** Seroprevalence of specific-*T. gondii* IgG and IgM among Tibetan sheep, yaks, cows, chickens, pigs, and horses in Qinghai.

Animals	Prefectures	No. of Tested *	Total IgG-Seropositive		Total IgM-Seropositive	
			Frequency	Prevalence (95% CI ^#^)	Frequency	Prevalence (95% CI ^#^)
Tibetan sheep	Haibei	135	49	36.3 (28.2–44.4)	11	8.1 (3.5–12.8)
	Hainan	92	56	60.9 (50.9–70.8)	31	33.7 (24.0–43.4)
	Haixi	90	28	31.1 (21.5–40.7)	10	11.1 (4.6–17.6)
	Yushu	112	30	26.8 (18.6–35.0)	16	14.3 (7.8–20.8)
	Guoluo	338	220	65.1 (60.0–70.2)	92	27.2 (22.5–32.0)
	Xining	40	14	35.0 (20.2–49.8)	10	25.0 (11.6–38.4)
	Haidong	60	51	85.0 (76.0–94.0)	15	25.0 (14.0–36.0)
	Huangnan	40	12	30.0 (15.8–44.2)	6	15.0 (3.9–26.1)
	Total	907	460	50.7 (47.5–54.0)	191	21.1 (18.4–23.7)
Yak	Haibei	20	4	20.0 (2.5–37.5)	4	20.0 (2.5–37.5)
	Hainan	20	2	10.0 (3.1–23.1)	0	0
	Haixi	20	3	15.0 (0.6–30.6)	0	0
	Yushu	20	4	20.0 (2.5–37.5)	1	5.0 (4.6–14.6)
	Guoluo	411	105	25.5 (21.3–29.8)	36	8.8 (6.0–11.5)
	Xining	119	14	11.8 (6.0–17.6)	3	2.5 (0.3–5.3)
	Haidong	20	1	5.0 (4.6–14.6)	0	0
	Huangnan	33	7	21.2 (7.3–35.2)	0	0
	Total	663	140	21.1 (18.0–24.2)	44	6.6 (4.7–8.5)
Cow	Haidong	205	38	18.5 (13.2–23.9)	31	15.1 (10.2–20.0)
Chicken	Xining	200	67	33.5 (27.0–40.0)	12	6.0 (2.7–9.3)
	Haidong	300	162	54.0 (48.4–59.6)	50	16.7 (12.4–20.9)
	Total	500	229	45.8 (41.4–50.2)	62	12.4 (9.5–15.3)
Pig	Xining	145	128	88.3 (83.0–93.5)	59	40.7 (32.7–48.7)
	Haidong	172	156	90.7 (86.4–95.0)	71	41.3 (33.9–48.6)
	Haixi	20	20	100.0 (100.0–100.0)	11	55.0 (33.2–76.8)
	Total	337	304	90.2 (86.4–92.9)	141	41.8 (36.6–47.1)
Horse	Huangnan	61	8	13.1 (4.6–21.6)	-	-
Total		2673	1179	44.1 (42.2–46.0)	469	18.0 (16.5–19.4)

* No.: Number; ^#^ 95% CI: 95% Confidence Interval.

**Table 3:** The double and single IgG and IgM seropositive samples among Tibetan sheep, yaks, cows, chickens, pigs, and horses in Qinghai.

Animals	Prefectures	No. of Tested *	No. of Positive Samples (%) *	Both IgG and IgM Seropositive		Single-IgG-Seropositive		Single-IgM-Seropositive	
				Frequency	Prevalence (95% CI ^#^)	Frequency	Prevalence (95% CI ^#^)	Frequency	Prevalence (95% CI ^#^)
Tibetan sheep	Haibei	135	50 (37.0)	10	7.4 (3.0–11.8)	39	28.9 (21.2–36.5)	1	0.7 (0.7–2.2)
	Hainan	92	64 (69.6)	23	25.0 (16.2–33.8)	33	35.9 (26.1–45.7)	8	8.7 (2.9–14.5)
	Haixi	90	29 (32.2)	9	10.0 (3.8–16.2)	19	21.1 (12.7–29.5)	1	1.1 (1.1–3.3)
	Yushu	112	36 (32.1)	10	8.9 (3.6–14.2)	20	17.9 (10.8–25.0)	6	5.4 (1.2–9.5)
	Guoluo	338	228 (67.5)	84	24.9 (20.2–29.5)	136	40.2 (35.0–45.5)	8	2.4 (0.7–4.0)
	Xining	40	19 (47.5)	5	12.5 (2.3–22.7)	9	22.5 (9.6–35.4)	5	12.5 (2.3–22.7)
	Haidong	60	52 (86.7)	14	23.3 (12.6–34.0)	37	61.7 (49.4–74.0)	1	1.7 (1.6–4.9)
	Huangnan	40	16 (40.0)	2	5.0 (1.8–11.8)	10	25.0 (11.6–38.4)	4	10.0 (0.7–19.3)
	Total	907	494 (54.5)	157	17.3 (14.8–19.8)	303	33.4 (30.3–36.5)	34	3.7 (2.5–5.0)
Yak	Haibei	20	5 (25.0)	3	15.0 (0.6–30.6)	1	5.0 (4.6–14.6)	1	5.0 (4.6–14.6)
	Hainan	20	2 (10.0)	0	0	2	10.0 (3.1–23.1)	0	0
	Haixi	20	3 (15.0)	0	0	3	15.0 (0.6–30.6)	0	0
	Yushu	20	4 (20.0)	1	5.0 (4.6–14.6)	3	15.0 (0.6–30.6)	0	0
	Guoluo	411	117 (28.5)	24	5.8 (3.6–8.1)	81	19.7 (15.9–23.6)	12	2.9 (1.3–4.5)
	Xining	119	15 (12.6)	2	1.7 (0.6–4.0)	12	10.1 (4.7–15.5)	1	0.8 (0.8–2.5)
	Haidong	20	1 (5.0)	0	0	1	5.0 (4.6–14.6)	0	0
	Huangnan	33	7 (21.0)	0	0	7	21.2 (7.3–35.2)	0	0
	Total	663	154 (23.2)	30	4.5 (2.9–6.1)	110	16.6 (13.8–19.4)	14	2.1 (1.0–3.2)
Cow	Haidong	205	56 (27.3)	13	6.3 (3.0–9.7)	25	12.2 (7.7–16.7)	18	8.8 (4.9–12.7)
Chicken	Xining	200	67 (33.5)	12	6.0 (2.7–9.3)	55	27.5 (21.3–33.7)	0	0
	Haidong	300	165 (55.0)	47	15.7 (11.6–19.8)	115	38.3 (32.8–43.8)	3	1.0 (0.1–2.1)
	Total	500	232 (46.4)	59	11.8 (9.0–14.6)	170	34.0 (29.8–38.2)	3	0.6 (0.1–1.3)
Pig	Xining	145	134 (92.4)	52	35.9 (28.1–43.7)	75	51.7 (43.6–59.9)	7	4.8 (1.3–8.3)
	Haidong	172	160 (93.0)	67	39.0 (31.7–46.2)	89	51.7 (44.3–59.2)	4	2.3 (0.1–4.6)
	Haixi	20	20 (100.0)	11	55.0 (33.2–76.8)	9	45.0 (23.2–66.8)	0	0
	Total	337	314 (93.2)	130	38.6 (33.4–43.8)	173	51.3 (46.0–56.7)	11	3.3 (1.4–5.2)
Horse	Huangnan	61	8 (13.1)	-	-	8	13.1 (4.6–21.6)	-	-
Total		2673	1258 (47.1)	389	14.9 (13.5–16.3)	789	30.2 (28.4–32.0)	80	3.0 (2.4–3.7)

* No.: Number; ^#^ 95% CI: 95% Confidence Interval.

**Table 4:** Analysis of the influence of altitude on the seroprevalence of *T. gondii* IgG and IgM antibodies and the distribution of *T. gondii* in Qinghai.

Animal\Altitude (m)		2000–3000	3000–4000	4000–5000	*p*-Value
Tibetan sheep	No. of tested	252	302	353	
	No. of IgG positive (%)	145 (57.5)	107 (35.4)	208 (58.9)	0.0012
	No. of IgM positive (%)	52 (20.6)	46 (15.2)	93 (26.3)	0.0005
Yak	No. of tested	179	251	233	
	No. of IgG positive (%)	22 (12.3)	62 (24.7)	56 (24.0)	0.0019
	No. of IgM positive (%)	7 (3.9)	8 (3.2)	29 (12.4)	0.0038
Chicken	No. of tested	450	50	0	
	No. of IgG positive (%)	199 (44.2)	30 (60.0)	-	0.2138
	No. of IgM positive (%)	40 (8.9)	22 (44.0)	-	<0.0001
Pig	No. of tested	337	0	0	
	No. of IgG positive (%)	304 (90.2)	-	-	-
	No. of IgM positive (%)	141 (41.8)	-	-	-

## Data Availability

Data is contained within the article.
